# A Systematic Review: The Effect of Cancer on the Divorce Rate

**DOI:** 10.3389/fpsyg.2022.828656

**Published:** 2022-03-09

**Authors:** Dominik Fugmann, Martin Boeker, Steffen Holsteg, Nancy Steiner, Judith Prins, André Karger

**Affiliations:** ^1^Medical Faculty, Clinical Institute of Psychosomatic Medicine and Psychotherapy, Heinrich-Heine-University-Düsseldorf, Düsseldorf, Germany; ^2^University Medical Center Rechts der Isar, School of Medicine, Institute of Artificial Intelligence and Informatics in Medicine, Technical University of Munich, Munich, Germany; ^3^Department of Medical Psychology, Radboud Institute of Health Sciences, Radboud University Medical Centre, Nijmegen, Netherlands

**Keywords:** cancer, oncology, psycho-oncology, divorce, separation, marriage, couple, spouse

## Abstract

**Introduction:**

Research on the impact of cancer on close relationships brings up conflicting results. This systematic review collects empirical evidence on the research questions whether a cancer diagnosis in general or the type of cancer affects the divorce rate.

**Materials and Methods:**

This systematic review was conducted according to the guidelines of the Cochrane Collaboration and the PRISMA statement. The following electronic databases were searched: Web of Science, Ovid SP MEDLINE, PsycINFO, PsyINDEX, CINAHL, ERIC. Risk of bias assessment was performed with the preliminary risk of bias for exposures tool template (ROBINS-E tool). The grading of methodological quality was assessed with the Newcastle-Ottawa Scale.

**Results:**

Of 13,929 identified records, 15 were included in the qualitative synthesis. In 263,616 cancer patients and 3.4 million healthy individuals, we found that cancer is associated with a slightly decreased divorce rate, except for cervical cancer, which seems to be associated with an increased divorce rate.

**Discussion:**

According to this systematic review, cancer is associated with a tendency to a slightly decreased divorce rate. However, most of the included studies have methodologic weaknesses and an increased risk of bias. Further studies are needed.

## Background

Divorce is a common occurrence around the world, with significant differences between countries. In 2019 there were 1.8 divorces in 1,000 residents in Germany (USA 2019: 2.7 divorces in 1,000 residents) (Centers for Disease Control and Prevention [CDC], 2021; Destatis Statistisches Bundesamt, 2021). A divorce can have harmful consequences: in addition to social and economic impacts, health can also be impaired (Amato, 2000; Sbarra et al., 2011; Sbarra, 2015; Leopold, 2018). A subgroup of divorced people shows significantly increased mortality as a result (Sbarra, 2015). For cancer patients, social and emotional support from close relationships are among the most protective factors (Aizer et al., 2013). In view of 18.1 million cancer diagnoses per year and an increasing tendency worldwide (International Agency for Research on Cancer [IARC], 2020), an effect of cancer on the divorce rate would be of considerable relevance.

Cancer leads to distress in patients but also in their partners in dyadic relationships (Hodges et al., 2005; Hagedoorn et al., 2008). In literature there is evidence that distress increases within one year after diagnosis (Sjövall et al., 2009). The long-term effects of cancer on relationships are less clear (Manne and Badr, 2008; Regan et al., 2012). Furthermore, it is uncertain if there are detrimental effects on the quality of the relationship that can lead to a divorce due to a failure to cope. The literature is inconsistent in this regard: on the one hand, some studies report no higher risk of divorce after a cancer diagnosis of one spouse (Dorval et al., 1999; Joly et al., 2002; Carlsen et al., 2007). On the other hand, some studies provide evidence of a higher risk of divorce after a cancer diagnosis (Kirchhoff et al., 2012; Song et al., 2014).

In addition to known factors such as age at marriage or number of children (Heaton, 1990), the type of cancer could also have an influence on the risk of divorce. In a cohort study, 46,303 patients from the Danish cancer registry were compared to 221,028 matched patients from a Danish administrative registry: A higher divorce rate was only found in patients with cervical cancer (Carlsen et al., 2007). Keeping in mind that interdisciplinary cancer treatment is now organized in cancer type-specific centers, interventions that target unmet needs like maintaining the partnership could be easily implemented in clinical pathways. Targeted support in maintaining the partnership could be provided by all healthcare professionals in oncology, ranging from the provision of information to interventions to improve the quality of the relationship.

This systematic review collects empirical evidence on the research questions whether cancer in general or specific cancer types have an effect on the divorce rate.

In the literature a wide range of definitions of “marriage” and “divorce” can be found. Sometimes the category “divorced” includes both separated and divorced patients (Karraker and Latham, 2015), sometimes cohabitating couples are declared as married and moving into separate places of residence is declared a divorce (Carlsen et al., 2007; Dinh et al., 2018). Yet cohabitating couples separate more often than married couples (Bouchard, 2006). Accordingly, a systematic investigation of divorces following a cancer diagnosis could entail the risk of confounding because the groups compared can be composed differently, so that in the present systematic review only legally married couples and official divorces are considered in order to maximize internal validity.

## Materials and Methods

This systematic review was conducted following a protocol that was developed according to the guidelines of the Cochrane Collaboration and the PRISMA statement (Higgins and Green, 2011; Moher et al., 2015).

### Objectives, Definitions, Inclusion, and Exclusion Criteria

The primary objective of this systematic review was to collect evidence in as a complete manner as possible, and to extract and synthesis it for changes in the divorce rate after a cancer diagnosis. The secondary objective was analyzing the collected evidence to determine whether diagnoses of different cancer types are associated with changes in the divorce rate.

In terms of the evidence, we only considered married couples for the review, which were defined as a couple relationship between two adults (aged ≥ 18 years), regardless of gender, who are officially married. We included cancer patients with solid or non-solid tumors of all organ systems, who were diagnosed during marriage. A diagnosis prior to marriage did not qualify for our review and such data was excluded. Healthy subjects or those with different types of cancer were studied as a comparison group while comparison groups with diseases other than cancer were excluded. The outcome examined in this review is the divorce rate. A divorce was defined as a certified separation of a former married couple. Studies which included the defined outcome but which were not necessarily restricted to this outcome were included: experimental and/or observational studies, randomized and non-randomized studies, prospective or retrospective cohort studies and descriptive studies. The following study types were excluded: qualitative studies, studies not presenting an outcome including commentaries, letters and editorials, studies not publicized in full-text and not-obtainable in full-text, studies only presenting marital status data within 6 months of the cancer diagnosis.

### Search Strategy and Sources

A search strategy was developed to perform a wide search. Before the final search was performed, the Web of Science search strategy was reviewed by a PhD-level information scientist using PRESS: Peer review of Search Strategies model (McGowan et al., 2016).

A MESH term search while testing the search strategy did not yield any additional hits, so it was removed in the final search strategy. The search terms used are listed in Supplementary Material.

To ensure the relevance of the data, only studies released later than 1999 were considered. Only publications in English or German were considered due to the language abilities of the authors.

The following electronic databases were searched on 1st April 2020, a search update was carried out on 3rd June 2021. All studies were retrieved based on that search.

- Web of Science (Web of Science Core Collection, BIOSIS Citation Index, BIOSIS Previews, Current Contents Connect, Data Citation Index, Derwent Innovations Index, KCI-Korean Journal Database, MEDLINE, Russian Science Citation Index, SciELO Citation Index, Zoological Record)
- Ovid SP MEDLINE
- APA PsycINFO
- PsyINDEX
- CINAHL
- ERIC.

A complete sample search in Web of Science can be found in Supplementary Material.

To complete the search the following procedures were performed (hand search and cited-reference searches):

Reviewing the reference lists of the included publications, contacting experts in the examined field of psycho-oncology to gather information about other publications or not-yet published works (i.e., doctoral theses), performing a search for trial- and review registries, performing a citation search in Web of Science to find publications citing the publications included in the review and searching the local library catalog (Heinrich-Heine University Düsseldorf) for further publications.

### Study Selection Criteria and Study Selection

**FIGURE 1:**
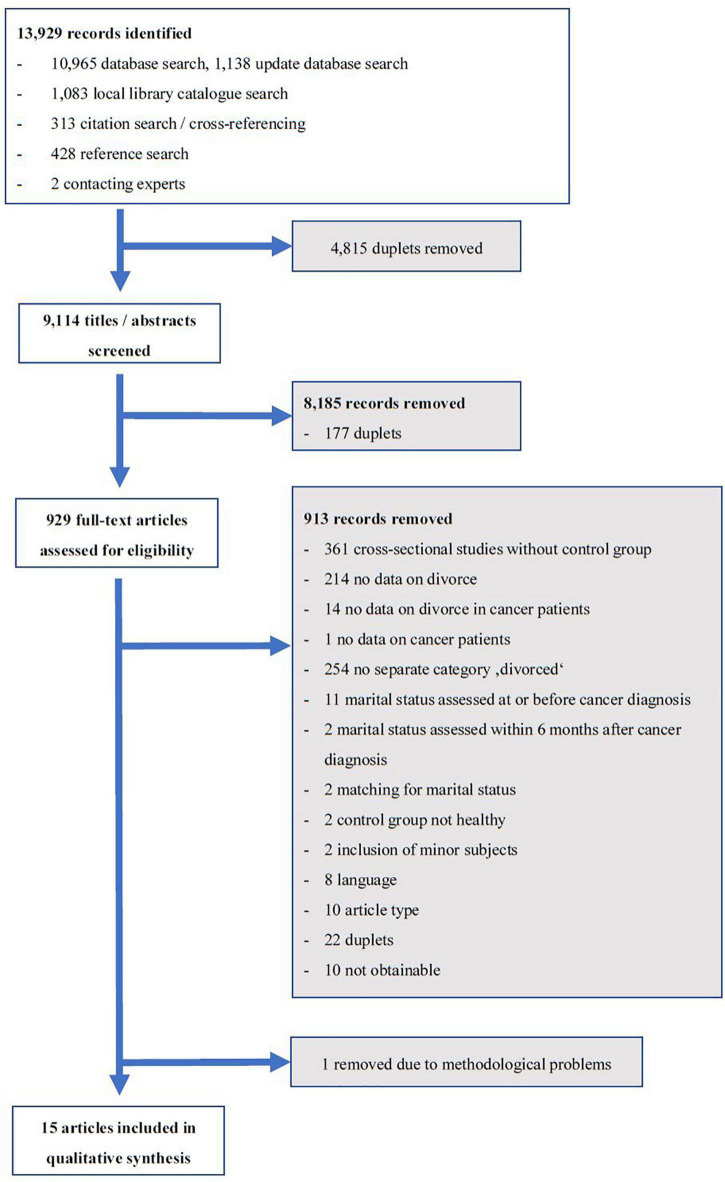
PRISMA flow chart.

Two authors (DF, SH or NS) independently categorized all discovered publications by title and abstract screening to determine whether these were to be included in, or excluded from, the review. If the classification remained unclear after abstract screening or the judgment was not unanimous, the full-text was obtained for a consensus-based decision of the two authors. All publications included at this point were obtained in full-text and reviewed by two authors (DF, SH or NS). Inclusion or exclusion of every publication was discussed by these authors. If no consensus could be achieved the publication in question was reviewed by another author (AK) who decided on inclusion and exclusion. We tracked all results in a Citavi Database (Citavi 6.3 2018). The selection process was recorded to create a PRISMA flow diagram (Figure 1).

### Data Extraction Procedures

All publications remaining after this procedure were independently registered in a standardized data extraction form by two authors (DF, SH, or NS). Discrepancies were discussed by at least two authors to reach consensus. Only those parts of the studies dealing with divorce and meeting the inclusion criteria were extracted and evaluated. The following domains were assessed: Source, methods, participants, independent variable, outcome, data analysis, risk of bias, methodological quality, results. A detailed description of the data sheet is available in the Supplementary Material.

### Risk of Bias Assessment

Two authors independently assessed the risk of bias and methodological quality in different domains (DF, SH). The overall risk of bias was graded as low, moderate, serious, or critical and the methodological quality was graded as low or high, in each case by two authors (DF, SH) independently. Discrepancies were discussed with another author (AK) to achieve consensus.

The risk of bias assessment of the interventional studies which were included was conducted according to the guidelines of the Cochrane Collaboration. Domains that were assessed are: Selection bias, performance bias, detection bias, attrition bias, reporting bias and other sources of bias.

The risk of bias for non-interventional studies was assessed with the preliminary risk of bias for exposures tool template (ROBINS-E tool) (Preliminary risk of bias for exposures tool template, 2020). The template is subdivided in “Preliminary considerations” and “Risk of bias assessment.” Preliminary considerations in terms of confounding areas regarding the divorce rate assessed for this review were: socio-demographics, marriage details, socio-economic status and country. Preliminary considered co-exposures for this review were co-morbidity, a previous cancer diagnosis, advanced cancer at diagnosis and impairing cancer therapy. Criteria used to determine the accuracy of exposure measurement were security of source and detailed description of cancer diagnoses. Factors to consider when evaluating health outcome assessment were: definition of “married,” definition of “divorce,” contamination of the category “divorced,” time between exposure and outcome assessed. Furthermore, study-specific confounding areas, co-exposures and criteria used to determine the accuracy of exposure measurement in the included studies were identified. The “Risk of bias assessment” includes the domains confounding, selection of participants, classification of exposures, departures from intended exposures, missing data, measurement of outcomes and selection of the reported result. The relative domain and finally the overall bias were graded in the categories low, moderate, serious, critical, and no information.

### Assessment of Methodological Quality

The methodological quality of non-randomized studies was assessed with the Newcastle-Ottawa Quality Assessment Scale (NOS), which includes the domains selection, comparability, and exposure/outcome (Wells et al., 2020). For cross-sectional studies, an adapted version was used (Herzog et al., 2013). The overall rating scale goes from zero to nine stars for each study (ten stars for cross-sectional studies). The methodological quality was defined as low if the overall rating was six stars or lower, or if studies were rated with only one star in the domains “selection of cases and controls or cohorts” or “assessment of outcome,” or if studies were rated with zero stars in any domain. The methodological quality of all other studies was defined as high.

### Synthesis of Extracted Evidence

The data was analyzed and classified according to the identified primary and secondary outcomes. In systematic qualitative synthesis, evidence was summarized. A summary of the methodology and results of each of the studies included was provided in table form. If possible, the ratio of divorced cancer patients to divorced couples without a cancer diagnosis was calculated for each study group. If groups of studies had similar designs, cohorts, and outcomes and furthermore had low risk of bias and a high methodical quality, a standard test of heterogeneity was planned: In case of low heterogeneity (<25%), studies were set to be included in a meta-analysis.

## Results

The search yielded 13,929 publications, of which 15 finally met the inclusion criteria (Figure 1). One study was removed from the analysis because it could not be interpreted due to methodological weaknesses and a critical “risk of bias” rating (Cheng et al., 2018).

Due to the small number of studies and the heterogeneity of the study designs and the investigated groups, no meta-analysis could be performed.

**FIGURE 2:**
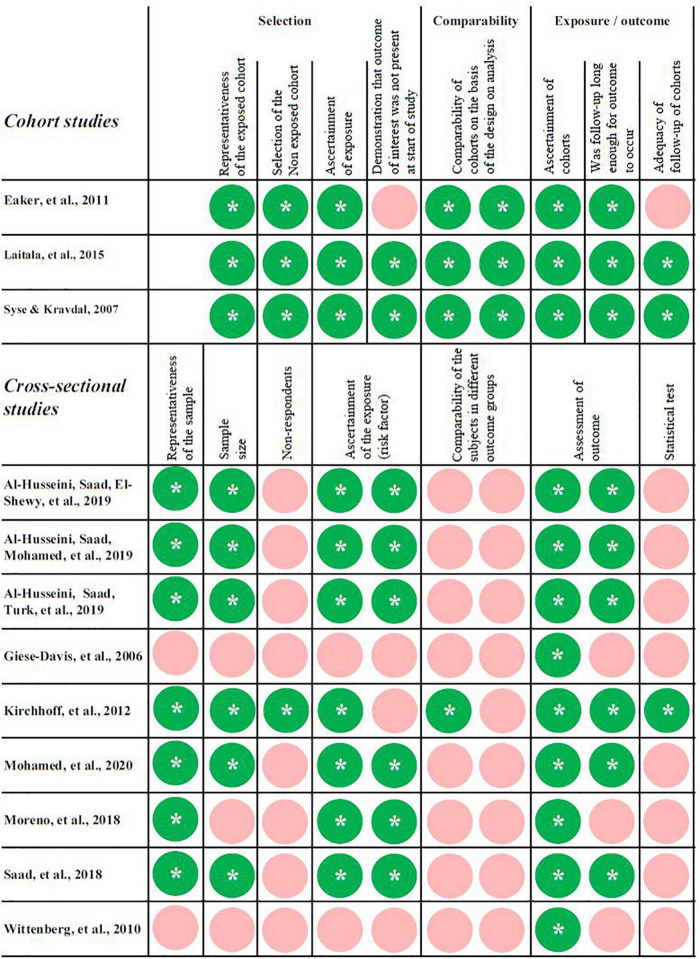
Rating of the methodological quality of the included cohort studies with the NOS (Wells et al., 2020). For the rating of the cross-sectional studies a modified version was used (Herzog et al., 2013). The overall rating scale goes from zero to nine stars (*) for each study (ten stars for cross-sectional studies).

The characteristics of the included studies are shown in Table 1, the corresponding rating of the methodological quality is illustrated in Figure 2.

**TABLE 1:** Characteristics of the included studies.

Author	Study design	Period after cancer diagnosis	Groups/number of participants	Malignancies	Risk of bias
Al-Husseini et al. (2019a)	Cross Sectional	7 months—41 years	Cancer *n* = 2,980 Comparison *n* = 46,587	Prostate cancer 35%, breast cancer 14%, colorectal cancer 9%, urinary bladder cancer 6%, melanoma 6%, endometrial tumor 5%, non-Hodgkin 4%, lung and bronchial cancer 3%, kidney cancer 3%, others 15%	Serious
Al-Husseini et al. (2019b)	Cross sectional	7 months—40 years	Cancer *n* = 30,516 Comparison *n* = 497,697	Prostate cancer 31.3%, breast cancer 20.8%, urinary bladder cancer 7.5%, corpus and uterus cancer 6.6%, lung and bronchus cancer 5.4%, skin excluding basal and squamous cancer 4.0%, non-Hodgkin 3.7%, others 20.8%	Serious
Al-Husseini et al. (2019c)	Cross sectional	≤41 years	Cancer *n* = 4,774 Comparison *n* = 59,821	Prostate cancer 31%, breast cancer 18%, colorectal cancer 11%, urinary bladder cancer 6%, endometrial cancer 4%, lung cancer 4%, melanoma 4%, kidney cancer 3%, non-Hodgkin 3%, others 16%	Serious
Bischofberger et al. (2009)	Descriptive	1 year	Breast cancer *n* = 108	Primary invasive breast cancer	Serious
Eaker et al. (2011)	Cohort	3–5 years	Breast cancer *n* = 4,761 Comparison *n* = 23,805	Primary invasive breast cancer	Moderate
Giese-Davis et al. (2006)	Cross sectional	Not available	Experienced *n* = 25 Newly diagnosed *n* = 29	Breast cancer	Serious
Kirchhoff et al. (2012)	Cross sectional	2–21 years	Young adult cancer *n* = 1,198 Comparison *n* = 67,063	Cervical cancer 41.9%, melanoma 12.4%, ovarian cancer 7.9%, thyroid cancer 5.3%, breast cancer 5.2%, endometrial cancer 4.2%, testicular cancer 2.8%, hodgkin disease 2.8%, non-Hodgkin 2.2%, leukemia 1.3%, bone cancer 1.1%, brain tumor 1.0%, other 11.9%	Moderate
Laitala et al. (2015)	Cohort	≤17 years	Breast cancer *n* = 3,225 Comparison *n* = 131,210	Early staged breast cancer (T1–4N0–3M0)	Moderate
Langer et al. (2010)	Descriptive	5 years	Hematological malignancy *n* = 121	Chronic myeloid leukemia 35.5%, acute leukemia 18.2%, myelodysplasia 13.2%, lymphoma 11.6%, solid tumor 14.0%, other 7.5%	Serious
Mohamed et al. (2020)	Cross sectional	7 months—41 years	Cancer *n* = 6,127 Comparison *n* = 115,303	Breast 56%, colorectal 14% melanoma 5%, thyroid 4%, non-Hodgkin 4%, kidney and renal pelvis 2%, urinary bladder 2%, lung and bronchus 2%, others 11%	Moderate
Moreno et al. (2018)	Cross sectional	Not available	Breast cancer *n* = 128 Prostate cancer *n* = 90 Colorectal cancer *n* = 70	Breast cancer 44.4%, colorectal cancer 24.3%, prostate cancer 31.3%	Serious
Odigie et al. (2010)	Descriptive	3 years	Breast cancer *n* = 81	Breast cancer (stages II and III)	Serious
Saad et al. (2018)	Cross sectional	7 months—41 years.	Cumulative cancer group *n* = 1,437 Cumulative comparison group *n* = 16,896	Prostate cancer 25%/43%, colorectal cancer 11%/10%, breast cancer 8%/5%, lung & bronchus cancer 8%/5%, urinary bladder cancer 4%/9%, non-Hodgkin 2%/3%, kidney cancer 2%/2%, melanoma 2%/4%, endometrial cancer 2%/2%, others 36%/16%	Serious
Syse and Kravdal (2007)	Cohort	≤ 27 years	Cancer *n* = 216,584 Comparison *n* = approx. 2.6 million	*Men (person-years of observation):* no cancer 23.3 million, testicular cancer 40,321, skin cancer 90,196, renal/bladder cancer 94,137, colorectal cancer 97,944, head/neck cancer 45,850, Morbus Hodgkin 9,546, prostate cancer 155,580, brain cancer 8,488, non-Hodgkin 21,408, endocrine cancer 9,315, leukemia 20,561, lung cancer 28,247, other cancer 44,670 *Women:* no cancer 23.5 million, cervical cancer 90,931, other gyn. cancer 125,011, breast cancer 242,228, skin cancer 82,586, endocrine cancer 30,533, colorectal cancer 73,447, Hodgkin 6,733, brain cancer 6,754 Renal/bladder cancer 24,893, non-Hodgkin 15,636, leukemia 12,033 Head/neck cancer 10,203, lung cancer 8,146, other cancer 24,993	Low
Wittenberg et al. (2010)	Cross sectional	Not available	Experienced *n* = 34 Newly diagnosed *n* = 39	Breast cancer	Serious

### General Cancer Diagnosis

Seven of the studies included present data on the primary question of whether cancer in general affects the divorce rate (Table 2).

**TABLE 2:** Estimate effects of cancer diagnoses in general on the divorce rate.

Author	Proportion divorced	Effect type	Estimate of effect
Al-Husseini et al. (2019a)	Cancer group 7.7% Comparison group 8.0%	Ratio	0.96
Al-Husseini et al. (2019b)	Cancer group 6.2% Comparison group 7.1%	Ratio	0.87
Al-Husseini et al. (2019c)	Cancer group 8.6% Comparison group 9.5%	Ratio	0.91
Mohamed et al. (2020)	Cancer group 9.5% Comparison group 10.2%	Ratio	0.93
Saad et al. (2018)	Cancer group 10.9% Comparison group 11.7%	Ratio	0.93
Kirchhoff et al. (2012)	Cancer group 14.1% Comparison group 9.6% US population 2009 Census 10.5%	RR	1.64 (95% CI 1.28–2.12)
Syse and Kravdal (2007)	Not available	OR	0.89 (95% CI 0.85–0.94)

Five very similar designed cross-sectional studies show marital status data on 739,599 subjects from the U.S. Surveillance, Epidemiology, and End Results database and compare two groups of patients with recent cancer diagnoses: Patients who already had a history of cancer before their recent second cancer diagnosis (*n* = 45,834) with those who have received their first cancer diagnosis ever (*n* = 736 304) (Saad et al., 2018; Al-Husseini et al., 2019a,b,c; Mohamed et al., 2020). In all five studies, the proportion of divorced patients in the previous cancer diagnosis group is marginally lower than in the comparison group with a ratio of divorce ranging from 0.87 to 0.96. Since all five studies primarily investigate other issues, the marital status data of the two groups was not adjusted in any way, so that a confounding must be assumed in several domains—the risk of bias was rated serious in all five studies. The methodological quality of the included parts of the studies is low (NOS 6/10 stars).

In a Norwegian registry study by Syse and Kravdal (2007) including 2.8 million subjects over an observation period of 27 years the odds ratio for divorce after a cancer diagnosis in already married subjects of 0.89 (95% CI 0.85–0.94) was calculated. The study was assessed with a low risk of bias and a high methodological quality (NOS 9/9 stars).

However, a cross-sectional study Kirchhoff et al. (2012) shows an increased risk ratio of 1.64 (95% CI 1.28–2.12) for divorce after a cancer diagnosis in one spouse. This study was conducted with 68,261 respondents, who were interviewed in 2009 in a telephone survey. The study was assessed with a moderate risk of bias and a high methodological quality (NOS 8/10 stars).

### Cancer Type

Ten of the studies included provided data on the effect of specific cancer types on the divorce rate (Table 3).

**TABLE 3:** Estimate effects of specific cancer types on the divorce rate.

Author	Proportion divorced	Effect type	Cancer type/estimate of effect
Kirchhoff et al. (2012)	Not available	RR	Cervical cancer 2.04 (95% CI 1.29–3.26)
Syse and Kravdal (2007)	Not available	OR	Men OR (95% CI):*^b^* Testicular cancer 1.05 (0.96–1.16) Skin cancer 0.96 (0.86–1.06) Renal/bladder cancer 0.88 (0.76–1.02) Colorectal cancer 0.80 (0.68–0.93) Head/neck cancer 1.00 (0.83–1.20) Morbus Hodgkin 1.02 (0.85–1.23) Prostate cancer 0.81 (0.67–0.98) Brain cancer 1.12 (0.92–1.37) Non–Hodgkin 0.80 (0.65–0.99) Endocrine cancer 1.01 (0.79–1.30) Leukemia 0.67 (0.50–0.89) Lung cancer 0.66 (0.49–0.88) Other cancer 0.83 (0.70–0.99) Women OR (95% CI):*^b^* Cervical cancer 1.36 (1.26–1.47) Other gyn. cancer 0.86 (0.78–0.96) Breast cancer 0.92 (0.85–0.99) Skin cancer 0.89 (0.80–0.98) Endocrine cancer 0.98 (0.85–1.12) Colorectal cancer 0.83 (0.69–0.99) Morbus Hodgkin 1.13 (0.91–1.40) Brain cancer 1.15 (0.92–1.44) Renal/bladder cancer 1.03 (0.80–1.32) Non-Hodgkin 0.80 (0.62–1.04) Leukemia 0.83 (0.59–1.17) Head/neck cancer 0.78 (0.53–1.15) Lung cancer 0.82 (0.54–1.24) Other cancer 0.82 (0.65–1.02)
Bischofberger et al. (2009)	Cancer group 0% *Divorce rate Germany 2009 2.3%^a^ (Destatis Statistisches Bundesamt, 2021)*	None	Breast cancer Not calculable
Eaker et al. (2011)	1 year prior: both groups 17.4%. After 3 years: cancer group 18.7% comparison group 19.4% After 5 years: cancer group 19.1% comparison group 19.7%	RR	Breast cancer After 3 years: 0.95 (95% CI 0.87–1.05) After 5 years: 1.00 (95% CI 0.90–1.10)
Laitala et al. (2015)	Cancer group 9.7% Comparison group 14.4%	HR	Breast cancer 0.98 (95% CI 0.80–1.18)
Giese-Davis et al. (2006)	Cancer group 4.0% Comparison group 13.8%	Ratio	Breast cancer 0.29
Wittenberg et al. (2010)	Cancer group 8.8% Comparison group 10.3%	Ratio	Breast cancer 0.85
Odigie et al. (2010)	After 3 years Cancer group 24.7% *Divorce rate Nigeria 2006 5.0%^a^ (Ntoimo and Akokuwebe, 2014)*	Ratio	Breast cancer 4.94
Moreno et al. (2018)	Breast cancer 14.8% Prostate cancer 7.7% Colorectal cancer 18.5% *Divorce rate USA 2018 7.7%^a^ (United States Census Bureau [USCB], 2020)*	Ratio	Breast cancer 1.92 Prostate cancer 1.00 Colorectal cancer 2.40
Langer et al. (2010)	After 5 years: Cancer group 7.3% *Divorce rate USA 2008 10.5%^a^ (United States Census Bureau [USCB], 2020)*	Ratio	Hematological malignancy 0.70

*^a^Data not part of the study. ^b^< 25% patients with a cancer diagnosis before marriage.*

#### Cervical Cancer

Two studies assessed divorce data on cervical cancer patients. Syse and Kravdal (2007) found an odds ratio for divorce after a cancer diagnosis of 1.36 (95% CI 1.26–1.47), Kirchhoff et al. (2012) found an increased risk of divorce after a cervical cancer diagnosis showing a risk ratio of 2.04 (95% CI 1.29–3.26).

#### Breast Cancer

Seven studies investigate breast cancer patients within the scope of the inclusion criteria of this systematic review:

In a German longitudinal, descriptive study Bischofberger et al. (2009) examined 108 patients for changes in their relationships one year after the initial diagnosis of breast cancer. During the observation period no divorce occurred. Information is lacking on many potentially confounding domains, so that the risk of bias was assessed as serious.

In a matched cohort study, Eaker et al. (2011) analyzed 4,761 breast cancer patients and 23,805 women matched by birth year and community. Time points were one year prior to the breast cancer patients’ calendar year of diagnosis, at the time of diagnosis, after three, and after 5 years. The risk ratio for divorce for the breast cancer survivors, adjusted for educational level, was 0.95 (95% CI 0.87–1.05) three years after diagnosis and 1.00 (95% CI 0.90–1.10) five years after diagnosis. The risk of bias was graded moderate, the methodological quality high (NOS 8/9 stars).

A total of 3,225 early stage breast cancer patients were compared to 131,210 healthy people in a Finnish registry study by Laitala et al. (2015). There was no significant difference in the divorce rate over a 10-year observation period. Overall, the adjusted hazard ratio for divorce in breast cancer patients was 0.98 (95% CI 0.80–1.18). The study was assessed with a moderate risk of bias and a high methodological quality (NOS 9/9 stars).

Two similar designed studies investigated a peer counseling intervention, in which newly diagnosed breast cancer patients (cumulative *n* = 68) were accompanied by breast cancer survivors with completed therapy, who were on average 52.20 and 59.56 months away from diagnosis (cumulative *n* = 59) (Giese-Davis et al., 2006; Wittenberg et al., 2010): Only baseline data in this study included data on divorce, which was extracted and assessed. A lower proportion of divorced patients was found in the more experienced group, compared to the newly diagnosed patients (4.0 vs. 13.8 and 8.8 vs. 10.3%). Accordingly, the divorce ratio is low in both studies. Not least because of the unadjusted group differences, this study was assessed with a serious risk of bias and a low methodological quality (NOS 1/10 stars).

A subgroup analysis in the Norwegian registry study by Syse and Kravdal (2007) showed, that the odds ratio for divorce is slightly lower for breast cancer patients. A limiting factor is that in this calculation, contrary to the review inclusion criteria, some of the subjects were diagnosed with cancer before marriage (< 25%).

Only one Nigerian descriptive study presented a high divorce rate in breast cancer patients within a follow-up period of three years compared to national data that was not part of the study (Odigie et al., 2010; Ntoimo and Akokuwebe, 2014). However, only 86 female patients after mastectomy were examined, most of whom lived in polygamous marriages. The risk of bias was rated serious in this study.

#### Hematologic Malignancies

Two studies provide divorce data on hematological malignancies:

Langer et al. (2010) examined 121 patients with hematological malignancies after hematopoietic stem cell transplantation, including patients with myelodysplastic syndrome (13.2%), which might not be considered as a malignancy. In a 5-year follow-up period the divorce rate was 7.3% (USA divorce rate 2008: 10.5%) (United States Census Bureau [USCB], 2020). The study was found to have a high risk of bias, partly because of an unclear proportion of subjects that was randomly assigned to an intervention to improve physical and cognitive limitations and manage emotional and family changes associated with hematopoietic stem cell transplantation.

A reduced odds ratio for divorce for leukemia and non-Hodgkin’s lymphomas was assessed by Syse and Kravdal (2007) only with respect to male patients. Again, a < 25% proportion of patients were diagnosed before marriage.

#### Colo-Rectal, Prostate, and Lung Cancer

In a cross-sectional study, data was divided into three groups: breast cancer (*n* = 128), prostate cancer (*n* = 90) and colorectal cancer (*n* = 70). There were no healthy controls (Moreno et al., 2018). The authors showed that the proportion of divorce was 14.8% for breast cancer patients, 7.7% for prostate cancer patients, and 18.5% for colorectal cancer patients. Since the groups were not adjusted and showed heterogeneity regarding socio-demographics, the risk of bias was assessed as serious and the methodological quality was assessed as low (NOS 4/10 stars).

Syse and Kravdal (2007) found a lower odds ratio of divorce in colorectal and prostate cancer patients compared to the healthy comparison group. A similar result was found in male but not female lung cancer patients.

## Discussion

Overall, according to six of the seven included studies on this question, there is evidence for a slightly decreased risk of divorce after a cancer diagnosis in general. The findings of Kirchhoff et al. (2012) differ from this conclusion, which is probably due to the following bias: a large proportion of patients in the cancer group suffered from cervical cancer, who were found to have a significantly increased risk ratio for divorce in the subgroup analysis. Furthermore, only young adult cancer survivors were examined: compared to older patients, younger patients more often are getting divorced after a cancer diagnosis (Syse and Kravdal, 2007). The fact that a cancer diagnosis does not increase the risk of divorce is supported by many studies, that apply a wider definition of separation in addition to official divorces (Dorval et al., 1999; Joly et al., 2002; Carlsen et al., 2007; Karraker and Latham, 2015).

Regarding the effect of a breast cancer diagnosis on the risk of divorce, most of the findings in the included studies are similar: a breast cancer diagnosis appears to have no or a decreasing effect on the risk for divorce. This finding is also consistent with other studies in the field that have examined other types of separations besides official divorces (Dorval et al., 1999; Carlsen et al., 2007).

Remarkably, the risk ratio or the odds ratio of divorce for cervical carcinoma patients is increased in the included studies (Syse and Kravdal, 2007; Kirchhoff et al., 2012). This coincides with the findings of Carlsen et al. (2007), who found an increased risk of divorce in a subgroup analysis for cervical cancer patients. Yet, in this study, the definition of divorce also included moving to different places of residence. Young people in particular are affected by this diagnosis, but the divorce rate is elevated among older individuals, as well (Syse and Kravdal, 2007). It is conceivable that infertility plays a role in this context. However, Syse and Kravdal (2007) did not find any influence of fertility on the odds ratio of divorce after a cancer diagnosis in their analysis, but there is evidence for a negative correlation between infertility distress and relationship satisfaction, which Ussher and Perz (2019) show in a survey of 693 women and 185 men with a cancer diagnosis. This infertility distress is not only persistent long term, but it is also associated with a higher rate of mental health disorders and psychosocial distress (Logan et al., 2019), which is an additional burden for the affected couple. Beyond that, depending on its stage and therapy, a diagnosis of cervical cancer may be associated with long-term changes in sexuality such as a tighter and shorter vagina, dyspareunia, and sexual worries (Lammerink et al., 2012; Wiltink et al., 2020). The resulting changes in sexual relationships are also a central issue for male partners of cervical cancer patients (Oldertrøen Solli et al., 2019). In a cross-sectional study with 113 cervical cancer patients a connection between sexual satisfaction and marital adjustment, partially moderated by body image, was found (do Rosário Ramos Nunes Bacalhau et al., 2020). It is also possible that the group of cervical cancer patients is composed differently than groups suffering from other cancer types because the risk of developing the disease is associated with early sexual intercourse and the number of sexual partners (International Collaboration of Epidemiological Studies of Cervical Cancer, 2009).

### Strengths and Limitations

Despite the large number of studies on the topic of separations or divorces after a cancer diagnosis, this is the first systematic review dedicated to this topic. The search strategy and inclusion criteria are very broad, including all countries and cultures, in order to fully reflect the current state of the literature. Thus, a very large number of studies could be found and screened in full-text. The focus on the outcome “divorce” instead of “separation” not only contributes to a high internal validity, but also allows a comparison of the results of the included studies with general divorce statistics.

However, as a result of this limitation exclusively to official divorces, only a few studies could be included. Some of these studies did not primarily investigate marital status in the context of cancer. Among other things, this has led to a more or less significant limitation of the methodological quality of these studies regarding divorce data. The significance of the present study is further diminished by the fact that no meta-analysis could be carried out because of the few and very heterogeneous studies included. Overall, the level of evidence provided by this systematic review is reduced due to the limitations mentioned. Further studies are needed to verify our results.

Time courses after a cancer diagnosis were not in scope of the present systematic review, although these proved to be quite relevant when reviewing the data: For example, Syse and Kravdal (2007) found an increased divorce rate within five years of a testicular cancer diagnosis, whereas the divorce rate is not increased after five years or overall.

### What Happens Within the Partnership?

The outcome “divorce” is too general to differentiate between specific positive and negative effects of a cancer diagnosis on a partnership. A well-researched model showing how couples deal with stressors such as a cancer diagnosis is that of dyadic coping (Bodenmann, 1995): Positive coping mechanisms like providing or accepting support maintain or even improve the relationship functioning (Traa et al., 2015). Negative coping behavior such as hiding worries can contribute to increased distress in the partnership (Kayser et al., 2007; Badr et al., 2010; Traa et al., 2015). Thus, there may be subgroups that are heavily distressed by a cancer diagnosis due to failed coping and whose marriages break up in the further course of the disease (Stephens et al., 2016), but which, in regard of the divorce rate, are balanced by subgroups where positive effects of a cancer diagnosis have led to an improved quality of relationships.

We must be cautious to interpret a divorce always as negative. For some couples it could be part of a developmental process. The negative impact of a stressful relationship should not be underestimated. Also being alone and having no social support might have a greater influence on health-related issues than being divorced (Metsä-Simola and Martikainen, 2013). After being divorced there are some cancer patients who will engage after a short time in a new relationship.

Future research has to investigate more closely the longitudinal processes within relationships dealing with cancer and relate individual factors and the dyadic process to health-related outcomes. An important task of such research is to identify risk factors and subgroups of patients and their families who need specific psychosocial support.

## Conclusion

Overall, we found evidence that cancer is associated with a slightly decreased divorce rate—an exception may be cervical carcinoma, which is associated with an increased divorce rate. The findings of the present study are limited by the heterogeneity and methodological weaknesses of most of the included studies. Thus, further research is needed, not only to validate the findings, but also to better understand the processes within the partnerships, with the aim of better adapting psychosocial support services to the vulnerable groups.

## Data Availability Statement

The original contributions presented in the study are included in the article/Supplementary Material, further inquiries can be directed to the corresponding author/s.

## Author Contributions

AK, DF, and MB contributed to conception and design of the study. DF and MB conducted the protocol and the searches. DF, SH, and NS performed the screening. DF and SH performed the data extraction and rating. DF wrote the first draft of the manuscript. All authors contributed to manuscript revision, read, and approved the submitted version.

## Conflict of Interest

The authors declare that the research was conducted in the absence of any commercial or financial relationships that could be construed as a potential conflict of interest.

## Publisher’s Note

All claims expressed in this article are solely those of the authors and do not necessarily represent those of their affiliated organizations, or those of the publisher, the editors and the reviewers. Any product that may be evaluated in this article, or claim that may be made by its manufacturer, is not guaranteed or endorsed by the publisher.

## Abbreviations

CI: confidence interval
HR: hazard ratio
NOS: Newcastle-Ottawa Quality Assessment Scale
OR: odds ratio
RR: risk ratio.
