# Supplementary material for: A Systematic Review: The Effect of Cancer on the Divorce Rate
Source: Front Psychol. 2022 Mar 9;13:828656. doi: 10.3389/fpsyg.2022.828656 (PMC8959852; doi:10.3389/fpsyg.2022.828656)
Supplement: Supplementary file 1 [file Data_Sheet_1.docx]

**A systematic review: The effect of cancer on the divorce rate**

**Supplemental material 1.** Search terms according to the PICOS scheme

| **PICOS scheme** | **Search terms** |
| --- | --- |
| **P** | ‘Spouse’ and all identified derivatives. ‘Marriage’ and all identified derivatives. ‘Couple’ and all identified derivatives (specific to the context). |
| **I** | ‘Cancer’ and all identified derivatives. |
| **C** | - |
| **O** | ‘Divorce’ and all identified derivatives. ‘Disruption’ and all identified derivatives (specific to the context). ‘Ending’ and all identified derivatives (specific to the context). ‘Quality of life’ was added to the search terms because many relevant publications contain marriage status data. |

**Supplemental material 2.** Sample search in Web of Science according to the PICOS scheme. Period: 1998 to present. Search date: 23.09.2019. Retrieval: 5051 records. Search terms according to the PICOS scheme

| PICOS scheme | Search terms |
| --- | --- |
| P | ((marri* OR marital* OR matrinomon* OR wed OR wedded OR  wedding OR conjugal* OR connubial*) OR  (wife OR bridal OR bride OR husband OR spous* OR "other half"  OR "better half") OR  ("interpersonal relation*" OR tie OR bond OR  ("quality of life")))  AND |
| I | ((cancer* OR neoplas* OR malign* OR tumour* OR tumor* OR  carcinom* OR sarcom* OR lymphom* OR leukemi* OR  leukaemi* OR blastom* OR metasta* OR anaplastic* OR CUP) OR  (MALT OR "multiple myelom*" OR hodgkin* OR waldenstrom* OR  waldenström*) OR  (astrozytom* OR paragangliom* OR schwannom* OR  meningiom*) OR  mesotheliom* OR  (HCC OR GIST OR insulinom* OR gastrinom* OR vipom* OR  glucagonom* OR somatostatinom* OR "signet-ring cell") OR  (pheochromo* OR phaeochromo* OR urotheliom* OR  seminom* OR teratom*) OR  (luteom* OR dysgerminom* OR epidermoid*) OR  (melanom* OR basaliom*) OR  (ewing* OR MFH OR "aggressive fibromatosis"))  AND |
| C |  |
| O | (divorc*  OR  ((((marri* OR marital* OR matrinomon* OR wed OR wedded  OR wedding OR conjugal* OR connubial*) OR (wife OR bridal OR  bride OR husband OR partner* OR spous* OR "other half" OR  "better half") OR ("interpersonal relation*" OR partner* OR  relation* OR couple OR tie OR bond OR union))  NEAR/20  (separat* OR split* OR dissociat* OR detach* OR disunit* OR  dissolut* OR segregat* OR sever* OR abondon* OR (call*  NEAR/3 quits)))  OR  (((wife OR bridal OR bride OR husband OR partner* OR spous*  OR "other half" OR "better half") OR ("interpersonal relation*" OR  partner* OR relation* OR couple OR tie OR bond ORunion))  NEAR/20  (disconne* OR (going NEAR/3 separate) OR (went NEAR/3  separate) OR (gone NEAR/3 separate)))  OR  (((marri* OR marital* OR matrinomon* OR wed OR wedded OR  wedding OR conjugal* OR connubial*) OR ("interpersonal  relation*" OR partner* OR relation* OR couple OR tie OR bond OR  union))  NEAR/20  (disjunction* OR fracturat*))  OR  (((marri* OR marital* OR matrinomon* OR wed OR wedded OR  wedding OR conjugal* OR connubial*) OR (wife OR bridal OR  bride OR husband OR partner* OR spous* OR "other half"  OR "better half"))  NEAR/20  ((break* NEAR/3 (up OR off OR down)) OR suspen*))  OR  ((marri* OR marital* OR matrinomon* OR wed OR wedded OR  wedding OR conjugal* OR connubial*)  NEAR/20  (disrupt* OR annul* OR terminat* OR disestablish* OR collap* OR  fail* OR shipwreck* OR miscarr* OR end* OR quit* OR ceas* OR  discontinu* OR stop* OR blow* OR destroy* OR (call* NEAR/3  off)))))  AND |
| S | (((clinical AND trial) OR clinical trials as topic OR clinical trial OR  random* OR random allocation OR therapeutic use) OR  (sensitiv* OR (sensitivity AND specificity) OR diagnos*) OR  (risk* OR "cohort studies" OR group*) OR  (incidence OR mortality OR "follow up studies" OR prognos* OR  predict* OR course*) OR  (predict* OR score* OR observ*)) |

**Supplemental material 3.** Data sheet

| **Category** | **Items** |
| --- | --- |
| Source | author, title, year of release, journal, language, source (search of databases of published studies, hand search or grey literature search) |
| Methods | study design, period, duration of study, details of the follow-up, number of study centers and locations, study setting |
| Participants | total number, inclusion criteria, exclusion criteria, age, gender, socio-demographics and -economics, county, details on matching for the control group, further details |
| Independent variable | total number of groups, details (malignancy or other) |
| Dependent variable / outcome | divorce / marital status: time points collected, divorce / marital status: time points reported |
| Data analysis | statistical technique, adjustment for confounding factors /marital status / divorce) |
| The Cochrane Collaboration’s tool for assessing risk of bias | selection bias (random sequence generation, allocation concealment), performance bias (blinding of participants and personnel), detection bias (blinding of outcome assessment), attrition bias (incomplete outcome data), reporting bias (selective reporting), other bias |
| ROBINS E-tool | confounding domains (socio-demographics: age, gender, number of children / marital status: age at marriage, marital duration, second or higher order marriage / socio-economic status: education, income / country), co-exposures with possible impact on study outcome (co-morbidity, previous cancer diagnosis, advanced cancer at diagnosis, impairing cancer therapy), accuracy of exposure measurement (security of source / detailed description of cancer diagnoses: number & proportion, cancer type, cancer stage / time of cancer diagnosis assessed), factors to consider (definition 'married', definition 'divorce', contamination of category 'divorced', time between exposure and outcome assessed), rating |
| Newcastle-Ottawa Quality Assessment Scale (NOS) | case control studies (‘Is the case definition adequate?’, representativeness of the cases, selection of controls, definition of controls, comparability of cases and controls on the basis of design or analysis, ascertainment of exposure, same method of ascertainment for cases and controls, non-response rate), cohort studies (representativeness of the exposed cohort, selection of the non-exposed cohort, ascertainment of exposure, demonstration that outcome of interest was not present at the start of study, comparability of cohorts on the basis of design or analysis, assessment of outcome, ‘was follow-up long enough for outcomes to occur?’, adequacy of follow up of cohorts), cross-sectional studies (representativeness of the sample, sample size, non-respondents, ascertainment of exposure (risk factor), comparability of the subjects in different outcome groups, assessment of outcome, statistical test), rating |
| Results | number of participants allocated to each group, missing participants, summary data for each group, estimate of effect with confidence interval or p value, subgroup analysis (according to cancer type) |
| Miscellaneous | funding score and notable conflicts of interest of study authors, key conclusions of study authors, references to other relevant studies, correspondence required, miscellaneous comments by review authors |
